# Efficacy of a spot-on combination product containing selamectin and sarolaner (Stronghold^®^ Plus) in the treatment of naturally occurring *Notoedres cati* infestations in cats

**DOI:** 10.3389/fvets.2025.1652148

**Published:** 2025-08-21

**Authors:** Stasia Borowski, Helena Berlamont, Thomas Geurden, Lina D’Hanis, Dhimiter Rapti, Dejan Cvejić, Oliver Suckstorff, Nikola Simović, Vickie L. King

**Affiliations:** ^1^Zoetis, Veterinary Medicine Research and Development, Zaventem, Belgium; ^2^Klinika Xhimi, Bulevardi Bajram Curri, Tirana, Albania; ^3^Argenta Munich, München, Germany; ^4^Zoetis, Veterinary Medicine Research and Development, Kalamazoo, MI, United States

**Keywords:** efficacy, selamectin, sarolaner, Stronghold^®^ Plus, cat, *Notoedres cati*, notoedric mange, feline scabies

## Abstract

Notoedric mange (also known as feline scabies) is a highly contagious and intensely pruritic dermatologic condition of cats caused by infestation with *Notoedres cati* mites. Previous publications provide evidence that topical selamectin, and more recently, topical selamectin + sarolaner is efficacious in the treatment of notoedric mange in cats. The study reported here was conducted to confirm the efficacy of a topically applied combination of selamectin and sarolaner (Stronghold^®^ Plus) in the treatment of notoedric mange in cats naturally infested with *N. cati.* Client-owned cats with clinical signs of notoedric mange and positive for live *N. cati* mites were enrolled and allocated randomly to treatment with either placebo (*n* = 10) or Stronghold Plus (*n* = 10). Treatment was administered on Days 0 and 30, and skin scrapings to detect live mites and assessment of the clinical signs of notoedric mange were conducted on Days 0, 30, and 60. The primary efficacy evaluation was based on the percent reduction in live mite counts in Stronghold Plus-treated cats compared to placebo-treated cats on Day 30. The parasitological cure (i.e., no live mites found) on Days 30 and 60 was also calculated for both treatment groups, as well as the improvement in clinical signs of notoedric mange. A single topical administration of Stronghold Plus provided a significant (*p <* 0.0001) and 100% reduction in live *N. cati* mites relative to placebo within 30 days. All placebo-treated cats harbored mites on Days 30 and 60, while none of the Stronghold Plus-treated cats had any live mites. Parasitological cure on Days 30 and 60 was thus 100% and significantly different than placebo (*p <* 0.0001). Clinical signs of notoedric mange improved in the Stronghold Plus-treated cats by Day 30, and no signs of notoedric mange were observed by Day 60. This study confirms that Stronghold Plus is highly effective in the treatment of notoedric mange in cats naturally infested with *N. cati.*

## Introduction

Notoedric mange (also known as feline scabies) occurs in cats and other felids throughout the world and is caused by infestation with *Notoedres cati* mites. Although the overall incidence of notoedric mange in cats is relatively rare, infested cats are highly contagious and local outbreaks often occur (1). The disease is most often acquired by direct contact with an infested cat and infestations can spread rapidly in groups of cats or kittens (2). Stray cats in poor condition seem to be more susceptible to infestation (3). Humans in direct contact with an infested cat are also susceptible, especially adolescents where transient dermatitis may occur (3).

All life-stages of the *N. cati* mite live in the host skin and do not survive off the host, but do crawl on the skin surface between molting which allows for transmission between hosts (4, 5). Larvae, nymph, and adult mites create burrows in the host skin, and burrows created by adult mites may extend deeper than the stratum corneum while nymphal burrows tend to be very shallow (6). In cats, the mites tend to burrow in the face and ears (6, 7). Diagnosis of notoedric mange is made by microscopic identification of *N. cati* mites in material obtained by skin scraping and mites are usually not difficult to find (8). The typical clinical signs of notoedric mange are characterized by intense pruritus, erythema, and local areas of hair loss on the margins of the ear and face. As the disease progresses, characteristic crusting, scaling, and hyperkeratosis develop. If the infestation remains untreated, secondary bacterial infections may occur, and cats can become severely debilitated and potentially die (6).

Treatment of cats with notoedric mange is directed at eliminating the *N. cati* mites. Due to the potential for rapid transfer of mites between cats, possible progression to severe debilitating disease, and the zoonotic potential, timely administration of miticidal treatment is recommended. Topical selamectin and the topical selamectin and sarolaner combination (Stronghold® Plus) are known to provide efficacy against *N. cati* mites (9–14), although neither active ingredient is currently available in any product approved for the treatment of notoedric mange.

The objective of the current study was to confirm that a topical application of a combination product containing both selamectin and sarolaner (Stronghold Plus) is effective in eliminating *N. cati* mites in cats with naturally occurring notoedric mange. This combination product is marketed under the tradename of Revolution® Plus in countries outside the EU and United Kingdom.

## Materials and methods

This placebo-controlled field study was conducted using client-owned cats at a single site in Europe (Albania) and complied with Good Clinical Practice Guidelines (15) and with the guidelines for Demonstration of Efficacy of Ectoparasiticides (16). The Study Protocol was reviewed and approved by the Zoetis Ethics Review Assessment team prior to implementation. Written informed consent from each cat’s owner was obtained for a cat to participate in the study. All personnel involved in making assessments of efficacy or safety were masked to treatment assignments. Day 0 was defined as the day treatment was first administered.

**Figure 1:**
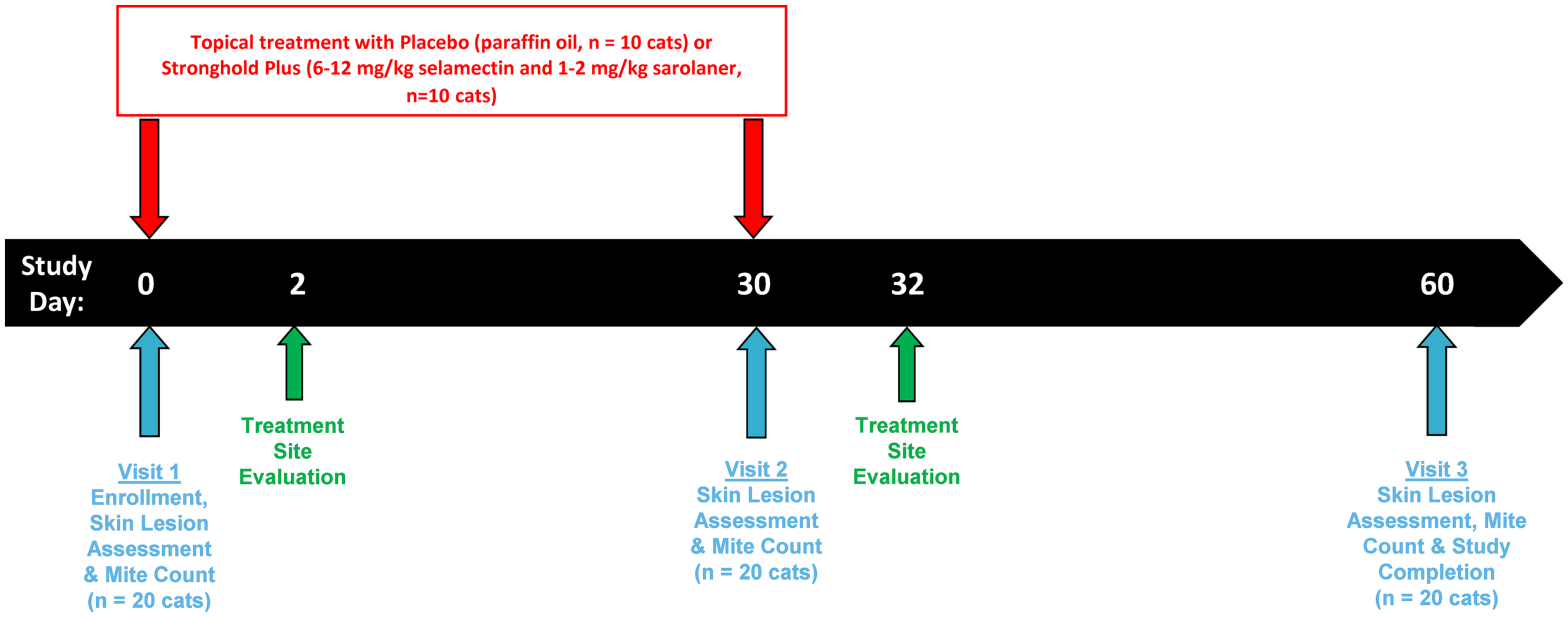
Graphical overview of the study design.

Enrolled cats had clinical signs of notoedric mange which included crusting, erythema, hair loss, papules, pruritus, pustules, and scaling. In addition, live *N. cati* mites (larvae, nymphs, or adults) were identified on skin scrapings prior to treatment. An overview of the study design is presented in Figure 1.

### Animals

The patient population was recruited from cats at least eight weeks of age and at least 1.25 kg bodyweight. There were no breed or gender restrictions, however pregnant females or cats intended for breeding were not eligible for enrollment.

Cats with a history of apparent reactions to products containing selamectin or sarolaner were excluded from enrollment, as were cats that had been treated with an ectoparasiticide with residual efficacy against *N. cati* mites at the time of enrollment.

In case of multi-cat households, the cat with the most severe clinical signs of notoedric mange was the primary patient (used for efficacy and safety evaluations) and all other cats in the same household were considered supplementary patients (used for safety evaluations only).

### Mite counts

Skin scrapings for detection of live *N. cati* mites were conducted on Days 0, 30, and 60. Deep scrapings were taken from the three body areas that showed the most severe or most likely clinical signs of infestation. Scrapings were performed over an area of approximately 1 cm^2^ to an approximately consistent depth. The collected material was transferred to mineral oil on a microscope slide and live *N. cati* mites (larvae, nymphs, and adults) and any eggs present were counted using 40X magnification.

### Clinical sign assessment

Clinical signs of notoedric mange were assessed before skin scraping and treatment on each mite count day. Each cat was thoroughly examined for skin lesions characteristic of notoedric mange including crusting, erythema, hair loss, papules, pruritus, pustules, and scaling. The severity of each clinical sign was scored as absent (no observable abnormality); mild (intensity/density is low and only a small area is affected); moderate (great intensity/density over a small area or of lesser intensity/density but affecting a large area); and severe (great intensity/density and covers a large area).

### Randomization

Primary cats were allocated to one of the two treatments according to a randomized complete block design. Cats were blocked based on order of enrolment in groups of two and each cat allocated randomly to treatment with either placebo or Stronghold Plus (selamectin + sarolaner) within the block. Any supplementary cats in the household received the same treatment as the primary cat.

### Treatment

Treatment was administered on Days 0 and 30 by the Dispenser (masked study personnel not involved in any efficacy and safety assessments). Doses were calculated based on the body weight of the cats collected immediately prior to each dosing.

Cats allocated to the control group were treated with 0.5 mL of paraffin oil. Cats allocated to the treated group were treated with commercial Stronghold Plus according to the approved product label directions which provided 6.0 to 12.0 mg/kg selamectin and 1.0 to 2.0 mg/kg sarolaner.

Treatments were applied topically at the base of the cat’s neck in front of the shoulder blades. The cat’s hair was parted and the treatment applied directly to the exposed skin. Each cat was observed for approximately one minute after dosing to confirm that there was no loss of the administered treatment due to run-off.

### Safety assessments

Physical examinations were performed by a suitably trained veterinarian on all cats prior to the first treatment administration on Day 0, and again on Days 30 and 60. All abnormal health events observed by the veterinarian during physical examinations or observed by the owner were recorded, as were any concomitantly administered medications. In addition, two days after each treatment administration (i.e., on Days 2 and 32) the treatment administration site was examined by a veterinarian for abnormalities including matting of the hair, spiking/stiff hair, wetness, white deposits, alopecia, erythema, and edema.

### Data analysis

All cats that received treatment were included in the safety assessments and only primary cats were included in the efficacy analyses. The primary cat in each household was the experimental unit and the primary endpoint was the total live *N. cati* mite count, which was calculated by adding together all live larvae, nymphs, and adults collected from the scraped sites on each cat.

The primary efficacy evaluation was determined based on the total live mite counts for the Stronghold Plus-treated cats relative to placebo on Day 30. Total live mite counts on Day 30 were analyzed using a general linear mixed model. The fixed effect was treatment, and the random effect was error. Treatment least squares means, standard errors, 95% confidence intervals, and minimums and maximums were calculated. Percent effectiveness of Stronghold Plus relative to placebo was calculated using least squares means based on the formula [(C-T)/C] x 100, where C = mean live mite count for the placebo group, T = mean live mite count for the treated group.

Secondary efficacy evaluations included comparisons of the total live mite counts on Day 60 compared to Day 0 for both treatment groups, and parasitological cure (no live mites found) on Days 30 and 60 for both treatment groups. Total live mite counts on Days 0 and 60 were analyzed by treatment group using a general linear mixed model. The fixed effect was time point, and the random effect was error. Treatment least squares means, standard errors, 95% confidence intervals, and minimums and maximums were calculated. Percent reduction on Day 60 compared to Day 0 was calculated for each treatment group using least squares means based on the formula [(Day 0-Day 60)/Day 0] x 100, where Day 0 = mean live mite count for Day 0, and Day 60 = mean live mite count for Day 60. Parasitological cure Yes/No on Days 30 and 60 was analyzed using Fisher’s Exact test by day as a generalized linear model failed to converge.

Mite egg counts were summarized by adding together all eggs collected from all scraped sites at each timepoint. Egg mite counts were not analyzed as they did not represent a measure of the presence of live mites at that time point.

## Results

### Patient demographics

Twenty primary cats (10 placebo and 10 Stronghold Plus) were enrolled and treated (Table 1). None of the primary cats lived in multi-cat households, therefore no supplementary cats were enrolled. All enrolled cats completed the entire study and data from all cats were included in all efficacy and safety evaluations.

**Table 1:** Demographics of cats enrolled in a European field study to evaluate the efficacy of Stronghold^®^ Plus in the treatment of natural *Notoedres cati* infestations in cats.

	Placebo (*n* = 10)	Stronghold Plus (*n* = 10)
Purebred	0	1
Non-purebred	10	9
Age mean (years)	2.9	3.0
Age range (years)	0.7–6.0	0.7–5.0
Bodyweight mean (kg)	2.7	3.1
Bodyweight range (kg)	1.7–3.5	1.5–5.3
Male	4	3
Female	6	7
Short haired	5	6
Medium haired	4	3
Long haired	1	1
Lives mostly indoors	0	0
Lives indoors and outdoors	9	5
Lives mostly outdoors	1	5

### Safety

No adverse health events were observed in any cat during the study. The only concomitant medications administered during the study were ketamine hydrochloride, xylazine hydrochloride, and atropine used for the sedation of one Stronghold Plus-treated cat for the skin scraping performed on Day 0.

There were no incomplete dosing events and thus all cats received a complete dose of either placebo or Stronghold Plus at the base of the neck in front of the shoulder blades on Days 0 and 30. Two days after each treatment, the administration sites for all placebo and Stronghold Plus-treated cats were normal with no signs of hair matting, spiking/stiff hair, wetness, white deposits, alopecia, erythema, or edema observed in any cat.

### Efficacy

**Figure 2:**
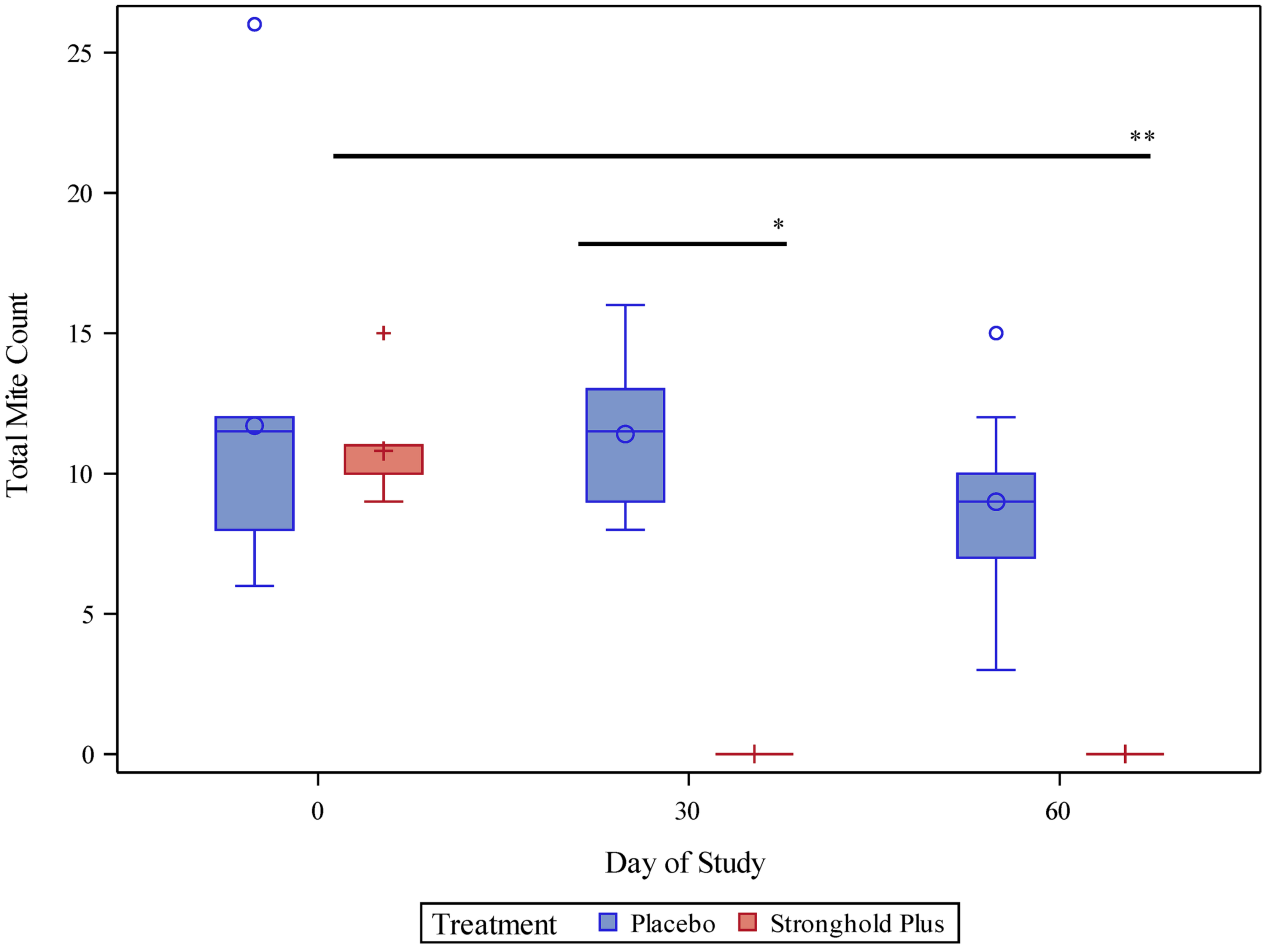
Pair-wise comparison of mite counts by treatment and study day. *Indicates a statistically significant difference between the Stronghold Plus-treated group and the placebo group on study day 30 (*p* < 0.0001). **Indicates a statistically significant difference between study day 60 and study day 0 within the Stronghold Plus-treated group (*p* < 0.0001).

On Day 0, live mite counts ranged from 6–26 (least squares mean 11.7) in the placebo group and from 9–15 (least squares mean 10.8) in the Stronghold Plus-treated group (Table 2 and Figure 2).

**Table 2:** Efficacy of Stronghold^®^ Plus in the treatment of natural Notoedres cati infestations in cats: *Notoedres cati* egg counts, and live mite counts, ranges, and percent reductions relative to pre-treatment and placebo.

	Study day		
	0	30	60
Placebo			
Number of cats	10	10	10
Mite counts			
Least Squares Mean	11.7^2^	11.4^1^	9.0^2^
Range	6-26	8-16	3–15
% reduction relative to pre-treatment	-	-	23.1
% of mite-free cats	0.0	0.0	0.0
Egg counts			
Geometric Mean	2.0	1.4	0.7
Range	1–4	0–3	0–2
Stronghold Plus			
Number of cats	10	10	10
Mite counts			
Least Squares	10.8^3^	0.0^1^	0.0^3^
MeanRange	9–15	0–0	0–0
% reduction relative to pre-treatment	-	-	100
% reduction relative to placebo	-	100	-
% of mite free cats	0.0	100	100
Egg counts			
Geometric Mean	2.1	0.0	0.0
Range	0–6	0–0	0–0

^1^ Stronghold Plus count significantly lower than placebo (*p* < 0.0001).

^2^ Day 60 count for placebo not significantly different than pre-treatment (*p* = 0.1962).

^3^ Day 60 count for Stronghold Plus significantly lower than pre-treatment (*p* < 0.0001).

On Day 30, all placebo-treated cats remained mite infested (range 8–16; least squares mean 11.4) while none of the Stronghold Plus-treated cats harbored any live mites. Thus, a single dose of Stronghold Plus provided a significant (*p <* 0.0001) reduction of 100% in live *N. cati* mites relative to placebo within 30 days after treatment.

On Day 60, all placebo-treated cats remained mite infested (range 3–15; least squares mean 9.0) while none of the Stronghold Plus-treated cats harbored any live mites. Relative to Day 0, live mite counts in the placebo group were reduced by 23.1% but were not significantly (*p* = 0.1962) lower than on Day 0. In contrast, live mite counts in the Stronghold Plus-treated group were reduced by 100% and were significantly (*p <* 0.0001) lower than on Day 0.

Live *N. cati* mites were found on all placebo-treated cats on Days 30 and 60, while no live mites were found on any Stronghold Plus-treated cat on either day. Parasitological cure (no live mites found) on Days 30 and 60 for the Stronghold Plus-treated group was thus 100% and significantly different from the placebo group on both days (*p <* 0.0001) (Table 3).

**Table 3:** Efficacy of Stronghold^®^ Plus in the treatment of natural *Notoedres cati* infestations in cats: percentage of Notoedres cati-free cats by study day.

	Study day		
	0	30	60
Placebo			
Number of cats	10	10	10
Percentage of mite free cats	-	0	0
Stronghold Plus			
Number of cats	10	10	10
Percentage of mite free cats	-	100 ^*^	100 ^*^

^*^Percentage of mite free cats treated with Stronghold Plus significantly different than placebo (*P* < 0.0001).

On Day 0, *N. cati* eggs were found on all placebo-treated cats (range 1–4) and on nine of the Stronghold Plus-treated cats (range 0–6) (Table 2). On Days 30 and 60, mite eggs were found on nine and six of the placebo-treated cats, respectively, while no eggs were found on any of the Stronghold Plus-treated cats on either Day.

On Day 0, the percentage of cats with clinical signs of notoedric mange in both treatment groups were identical with 10% of cats having pustules, 70% having papules, and 100% having crusting, erythema, hair loss, pruritus, and scaling (Table 4). In the placebo group the clinical signs remained stable throughout the study with 30% of cats having pustules, 80% having papules, and 100% having crusting, erythema, hair loss, pruritus, and scaling on Day 30, and 40% of cats having pustules, 80% having papules, and 100% having crusting, erythema, hair loss, pruritus, and scaling on Day 60. In the Stronghold Plus-treated group clinical signs improved with no cats having crusting, papules, or pustules, 10% having erythema and scaling, 20% having pruritus, and 70% having hair loss on Day 30, and no clinical signs of notoedric mange were present in any Stronghold Plus-treated cat on Day 60.

**Table 4:** Efficacy of Stronghold^®^ Plus in the treatment of natural *Notoedres cati* infestations in cats: percentage of cats with each clinical sign by study day.

Clinical sign	Study day		
	0	30	60
Placebo (*n* = 10)			
Crusting	100	100	100
Erythema	100	100	100
Hair Loss	100	100	100
Papules	70.0	80.0	80.0
Pruritus	100	100	100
Pustules	10.0	30.0	40.0
Scaling	100	100	100
Stronghold Plus (*n* = 10)			
Crusting	100	0.0	0.0
Erythema	100	10.0	0.0
Hair loss	100	70.0	0.0
Papules	70.0	0.0	0.0
Pruritus	100	20.0	0.0
Pustules	10.0	0.0	0.0
Scaling	100	10.0	0.0

## Discussion

The results of this study confirm that a single topical administration of Stronghold Plus at the approved dose range is highly efficacious in the treatment of notoedric mange in cats naturally infested with All cats treated with Stronghold Plus were mite-free within 30 days after the first treatment administration and all clinical signs of notoedric mange had completely resolved within 60 days after the first treatment. The excellent efficacy of Stronghold Plus observed in the current study is consistent with the high level of efficacy previously reported (9–14).

Prior to the first treatment on Day 0, *N. cati* eggs were found on cats in both treatment groups. On Days 30 and 60, mite eggs continued to be found on cats in the placebo group, while no eggs were found on any cat in the Stronghold Plus-treated group. Given the relatively short two-week lifecycle for *N. cati* (5), these results suggest that a single treatment with Stronghold Plus interferes with the *N. cati* lifecycle thus decreasing or even eliminating disease progression and transmission in cats.

Selamectin, a macrocyclic lactone endectocide, has been commercially available as Stronghold®/Revolution® for more than 20 years as a topically applied product for the prevention of heartworm disease, the treatment of roundworms and hookworms, the treatment and prevention of flea infestations, and the treatment of ear mite infestations in cats (17, 18). Selamectin alone provided little efficacy against ticks in cats, and therefore a combination product that combined selamectin with sarolaner (Stronghold Plus/Revolution Plus) was introduced. Sarolaner is an isoxazoline ectoparasiticide and as such provides broad-spectrum efficacy against fleas, ticks and mites (19); therefore both selamectin and sarolaner contribute to the ectoparasiticide efficacy provided by Stronghold Plus, although through different mechanisms of action (20–23). The excellent efficacy provided by Stronghold Plus in the treatment of cats with feline scabies caused by *N. cati* mites is therefore not unexpected given that both of the active ingredients have each alone demonstrated efficacy in the treatment of canine scabies caused by *Sarcoptes scabiei* mites (22, 23). Both *N. cati* and *S. scabiei* mites belong to the *Sarcoptidae* family, and the life cycles, biology, and pathology are very similar (24), with both being small parasitic mites for which the larvae, nymphs, and adults all burrow and create tunnels through the superficial layers of the epidermis. This burrowing through the skin damages keratinocytes resulting in cytokine release which causes cutaneous inflammation and the clinical signs typical of notoedric mange (25).

## Conclusion

In cats naturally infested with *N. cati*, a single topical dose of Stronghold Plus at the commercial dose range resulted in elimination of mites within 30 days after treatment, and in resolution of all clinical signs within 60 days after treatment, thus confirming that Stronghold Plus is highly effective in the treatment of notoedric mange in cats.

## Data Availability

The original contributions presented in the study are included in the article/supplementary material, further inquiries can be directed to the corresponding author/s.
